# Acceptability of a Pilot Digital Longitudinal Consultation Clinical Placement for Early‐Year Medical Students, Tutors and Patients

**DOI:** 10.1111/tct.70526

**Published:** 2026-09-15

**Authors:** Tina Huang, Anita Laidlaw, Rachel Falconer, Rona Patey

**Affiliations:** ^1^ University of Aberdeen Aberdeen UK

**Keywords:** acceptability, Digital Consultation Clinical Placement, medical education, medical students, patient‐centred care, remote consultations

## Abstract

**Introduction:**

The prevalence of remote consultations and the use of electronic health records necessitate their meaningful integration into medical education. Remote consultations may offer medical students valuable opportunities for longitudinal patient interactions that complement traditional clinical learning. In this regard, the University of Aberdeen piloted the Digital Consultation Clinical Placement (DCCP), which aimed to equip early‐year medical students with essential skills in carrying out remote consultations, conducting follow‐up consultations and practising medical documentation. This study aimed to evaluate the DCCP's acceptability and explore its associated challenges and opportunities.

**Methods:**

Acceptability of this novel placement was evaluated using questionnaires grounded in the theoretical framework of acceptability (TFA). Questionnaire data from 15 students, 4 tutors and 14 patient volunteers were analysed using descriptive statistics. Qualitative focus groups underwent thematic analysis to generate understanding of the challenges and opportunities of this innovative placement.

**Results:**

At least 50% of stakeholders selected *agree* or *strongly agree* in the questionnaire responses across all TFA categories. Qualitative findings suggest that remote consultations could help achieve the benefits of longitudinal patient contact, and exposure to authentic digital healthcare technology could impact students' professional identity.

**Conclusion:**

The DCCP was acceptable across all stakeholder groups, with a shared belief that it successfully achieved its intended purpose but with challenges and perceived opportunities for stakeholders, which should be considered in future placements. With further refinement, early exposure to this placement may support the development of patient‐centred care skills and better prepare students for technology‐enabled healthcare practice.

## 1 Introduction

In 2020, the COVID‐19 accelerated the widespread adoption of remote consultations [1], including telephone, video conferencing, online e‐consultation platforms, email and other web‐based asynchronous text‐based communication channels [2]. As these consultations are predicted to continue, they should be integrated into medical education [3]. Armstrong et al. [4] report that remote consultations provide distinct educational advantages: They enhance student autonomy, promote participation and facilitate the development of history‐taking and clinical reasoning skills. Sartori et al. [5] highlight that they require distinct skills, with inexperience limiting their effective use, which underscores the need to equip medical students with the skills and confidence to deliver care via this modality.

Digital health technologies, including electronic health records (EHRs), have transformed documentation and data access. Most EHR‐related education focuses on clinical clerkships, with limited training for early‐year students [6]. Beyond patient care, EHRs support legal documentation, research and quality improvement, reinforcing the need for training in remote consultations and EHR use.

Continuity of care fosters trusting patient–student relationships, enabling students to advocate effectively for patients and contribute to improving patient outcomes [7]. Rising medical student intake in the United Kingdom [8, 9] and sustained National Health Service pressures increasingly limit early‐year clinical placements, requiring reconsideration of traditional models.

Despite the widespread clinical use of remote consultations, longitudinal patient interactions and EHRs, evidence on their integration in medical education is limited. Integrating these approaches may support authentic learning—defined as acquiring knowledge and skills in contexts that mirror real‐world practice—which can enhance higher order thinking and preparedness for managing complex and chronic conditions [10, 11].

During 2024–2025, the University of Aberdeen piloted the Digital Consultation Clinical Placement (DCCP) with early‐year medical students. The DCCP offers an adjunct to traditional clinical training, face‐to‐face clinical placements, by allowing students to remotely consult patients, conduct follow‐up consultations and practice medical documentation through an EHR.

This study evaluates the acceptability of the DCCP among students, clinical tutors and patient volunteers and identifies the challenges and opportunities with its implementation for the organisers, students, patient volunteers and tutors.

## 2 Methods

### 2.1 DCCP

**FIGURE 1:**
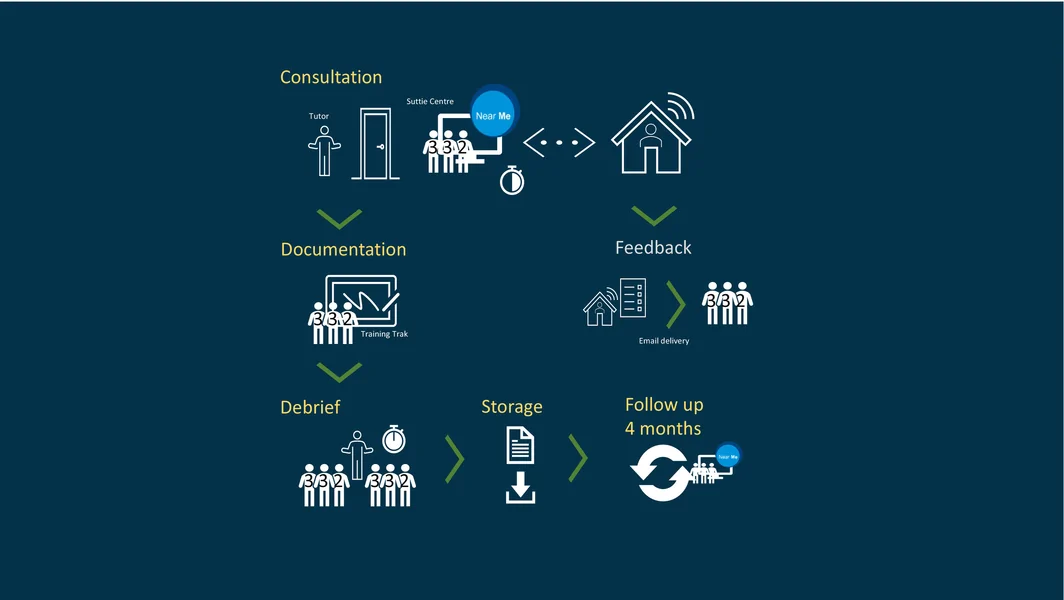
Structure of the Digital Consultation Clinical Placement (DCCP).

During the DCCP (Figure 1), medical students conducted independent consultations with patient volunteers via Near Me (a digital healthcare service used in Scotland that enables patients to access clinical consultations remotely), in collaboration with peers. A random student subsample participated in the pilot study.

Each group comprising two third‐year and one second‐year medical student completed an initial consultation and follow‐up with the same patient volunteer 4 months later. Patient volunteers had long‐term condition(s), and medical students explored how the condition(s) affected their lives and families, focusing on the impacts on daily functioning, including employment, social participation and routine activities. Medical students were located at the University of Aberdeen, while patient volunteers joined from home. Consultations lasted 30 min and were not recorded.

Each group produced a structured consultation summary of the patient encounter, either using the TrakCare system (an EHR system widely used in Scotland) or in written format. This occurred in a nonlive environment; no real patient records were accessed, and no patient‐identifiable information was recorded.

Subsequently, two groups participated in a 1‐h tutor‐led debrief for guided reflection, feedback and learning‐outcome discussion. After each consultation, patient volunteers completed an anonymous feedback questionnaire evaluating the students' consultation skills. This feedback supported student learning and reflection. Appendix S1 provides a DCCP replication framework.

### 2.2 The Theoretical Framework of Acceptability (TFA)

**FIGURE 2:**
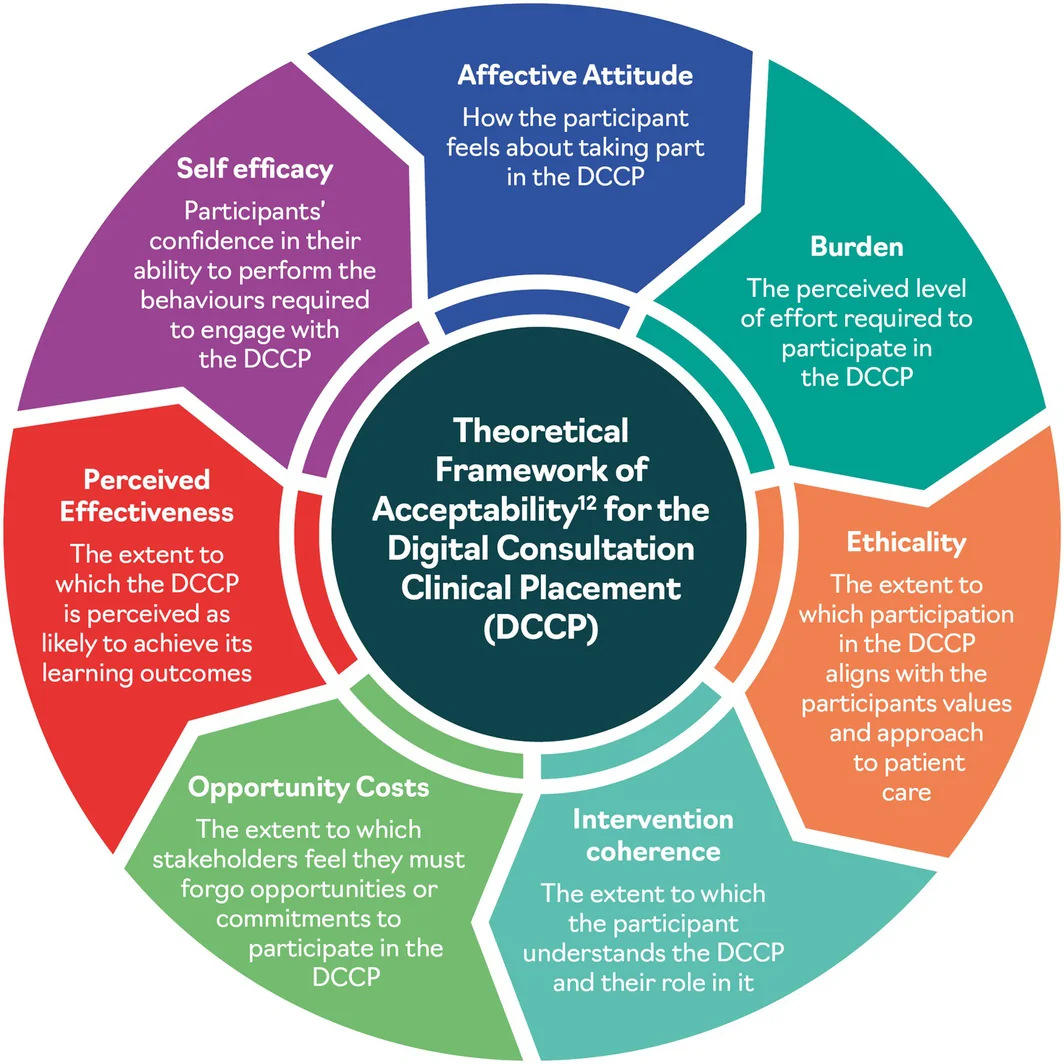
Modified theoretical framework of acceptability adapted for the DCCP, comprising seven components constructs [12].

The TFA provides a structured, evidence‐based approach to evaluating the acceptability of healthcare interventions from the perspectives of both recipients and providers [12]. This framework has been used to assess acceptability in digital healthcare interventions [13, 14] and for e‐training for aged care staff [15]. In the absence of a medical education‐specific acceptability framework, we adopted the TFA (Figure 2) because it accommodates patients, recognises patient engagement and has been applied in healthcare education interventions. Use of the TFA ensured consistency across questionnaires for all participant groups. Therefore, we designed the questionnaires based on the TFA [12] to assess the acceptability of the DCCP. In addition, we developed a global rating question as to whether student participants perceived the DCCP prepared them for practice. Single‐item global rating scales have been found to be valid in health research [16].

A pragmatist mixed methods approach, which emphasises participant perspectives and real‐word applicability [17], guided the study. Quantitative questionnaire data assessed DCCP acceptability, while qualitative focus group data provided deeper insights into the underlying mechanism driving that and identified the impact, challenges and opportunities [18].

### 2.3 Reflexivity

The researchers practised reflexivity throughout, recognising that their health professions education experience shaped the study. T.H., the DCCP lead researcher and designer, is a general practitioner and senior clinical lecturer experienced in teaching and evaluating educational initiatives. R.F. has a PhD, a surgical background and education research experience. A.L. is an experienced medical education researcher with no clinical background.

To enhance credibility, R.F. recruited participants and facilitated the focus groups, reducing potential coercion and bias given T.H.'s DCCP design role. A.L. did not collect data but reviewed, interpreted and analysed data, providing an independent medical education perspective. The research team met regularly and engaged in ongoing reflection throughout the study from their varying perspectives, enhancing trustworthiness of findings.

### 2.4 Recruitment and Sample Sufficiency

Eligible participants included (a) 40 medical students (10 second‐year and 30 third‐year students) who attended both sessions of the DCCP (we randomly selected a subsample of the whole cohort to participate in the pilot DCCP), (b) five clinical tutors who participated in the DCCP and (c) 16 patient volunteers who participated in either both consultations (*n* = 14) or only the second consultation (*n* = 2). All eligible participants were invited by email after the second session of the DCCP.

The research had a focused aim, was grounded in the TFA and was designed to evaluate a specific teaching project in which participants all experienced. Data collection and analysis prioritised an in‐depth exploration of participants perspectives rather than a broader cross‐case comparison [19]. Open text questions in the questionnaire and focus group data collection methods provided more opportunity for in‐depth review across sources. R.F., who conducted the focus groups, was an experienced researcher, further strengthening information power via rich data. Together, these factors supported a small sample size for meaningful findings.

### 2.5 Data Collection

Participants completed online questionnaires using Research Electronic Data Capture. Separate questionnaires were designed for medical students, patient volunteers and tutors. The data comprised responses using a 5‐point Likert scale and dichotomous (‘yes’/‘no’) questions based on the TFA [12]. Participation was voluntary and anonymous. Negatively worded questions were intentionally incorporated within the questionnaire to encourage participants to remain attentive and reflective while completing the survey.

Focus groups were conducted online, recorded in both video and audio formats using Microsoft Teams and transcribed via Microsoft Stream. Focus group questions were guided by the TFA (Appendix S1). Transcripts were verified against the recordings for accuracy, and participants were assigned generic pseudonyms to maintain anonymity.

### 2.6 Data Analysis

Quantitative questionnaire data were summarised using descriptive statistics [20]. Focus group data were analysed using thematic analysis to identify and report recurring patterns, making it suitable for understanding shared experiences, thoughts and behaviours [21, 22]. T.H. and R.F. initially reviewed the focus group data, familiarised themselves with the dataset and independently developed initial themes. Following iterative discussion among the researchers, common themes were established and reviewed, resulting in additional themes being added. T.H., R.F. and A.L. reviewed the data and the themes back and forth to ensure they aligned. Together, the researchers decided the final themes and reworded the final definitions to clarify the participants' experiences.

### 2.7 Ethics Approval

This study was approved by the School of Medicine, Medical Sciences and Nutrition Ethics Review Board at the University of Aberdeen (Reference ID 4574365).

## 3 Results

### 3.1 Sample

Fifteen students (38% of the potential sample) completed the student questionnaire, of which nine were third‐year and six were second‐year students. Four tutors (80%) completed the tutor questionnaire, and 14 patient volunteers (88%) completed the patient–volunteer questionnaire. Two focus groups were conducted: one comprising three third‐year students (10% of participants) and the other comprising two tutors (40% of participants). The use of a third‐year only focus group was justified by questionnaire findings, where second‐year students showed consistently more agreement across categories, suggesting limited variability for qualitative exploration. In contrast, third‐year students responses were more diverse, offering greater potential for in‐depth insight. Additionally, no second‐year students volunteered, resulting in a smaller but purposive sample.

### 3.2 Questionnaires

**FIGURE 3:**
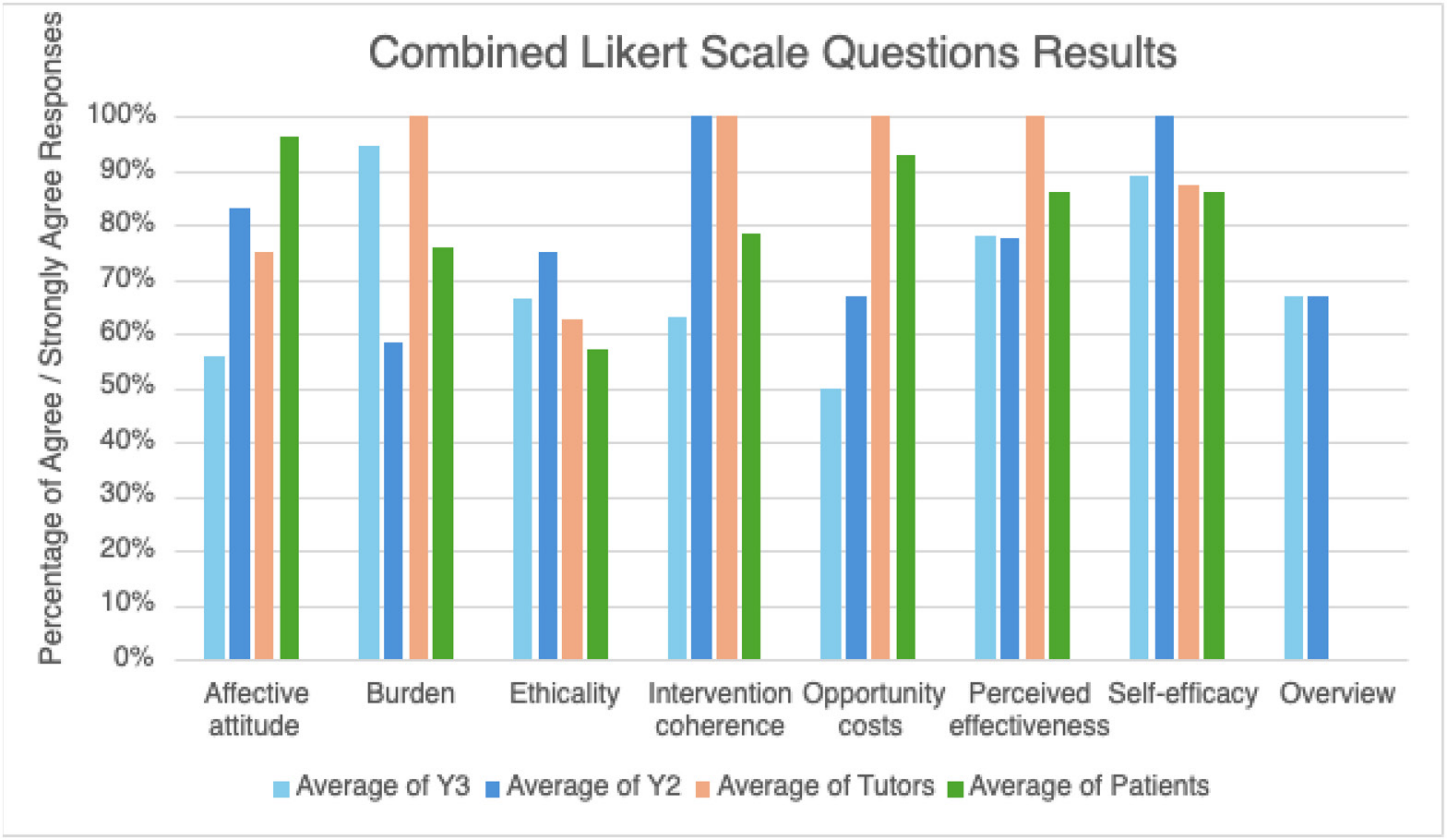
Combined Likert‐scale response data from student, tutor and patient questionnaires.

Figure 3 presents the combined questionnaire results of the Likert‐scale questions, categorised according to the TFA. In all categories, the percentage of stakeholders who responded with *agree* or *strongly agree* did not fall below 50%. Self‐efficacy, perceived effectiveness in achieving the placement aims and collective understanding had the highest average agreement across all stakeholders, at 91%, 86% and 86%, respectively. Additionally, 86% of patient volunteers answered that the remote DCCP was as effective as face‐to‐face contact.

**FIGURE 4:**
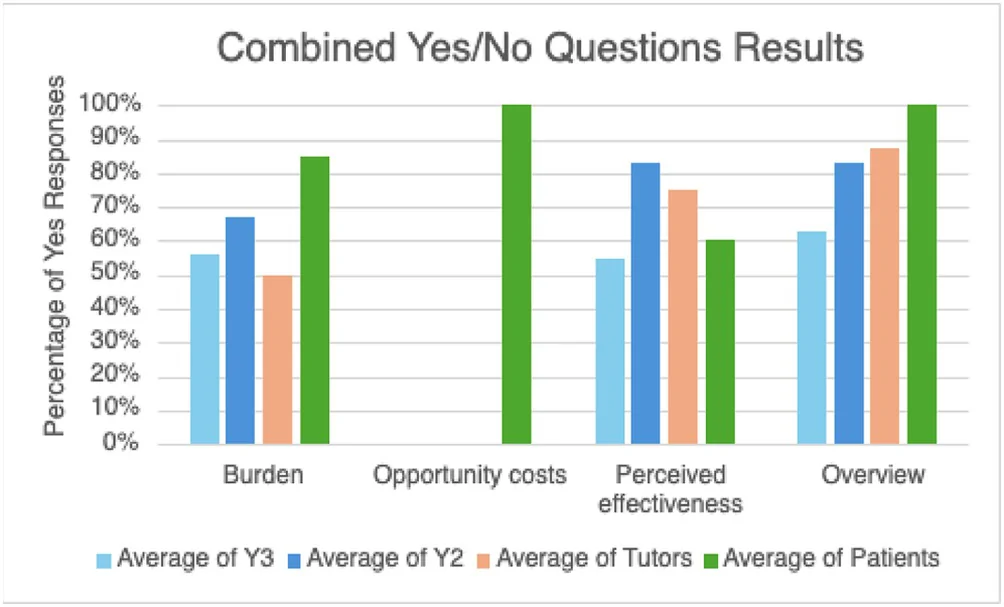
Combined dichotomous response data from student, tutor and patient questionnaires.

Certain questions were presented in a dichotomous format rather than using a Likert scale (Figure 4). The percentage of stakeholders who responded ‘yes’ did not fall below 50% in any category, with second‐year students demonstrating greater acceptance than third‐year students. All the stakeholder groups deemed the DCCP as acceptable.

Among medical students, 67% agreed or strongly agreed that the DCCP prepared them for future clinical placements, and 78% agreed or strongly agreed with its perceived effectiveness (Table S1). All tutors and patient volunteers expressed willingness to participate in future DCCPs as regular tutors or patient volunteers (see Tables S2 and S3, respectively).

### 3.3 Focus Groups

**FIGURE 5:**
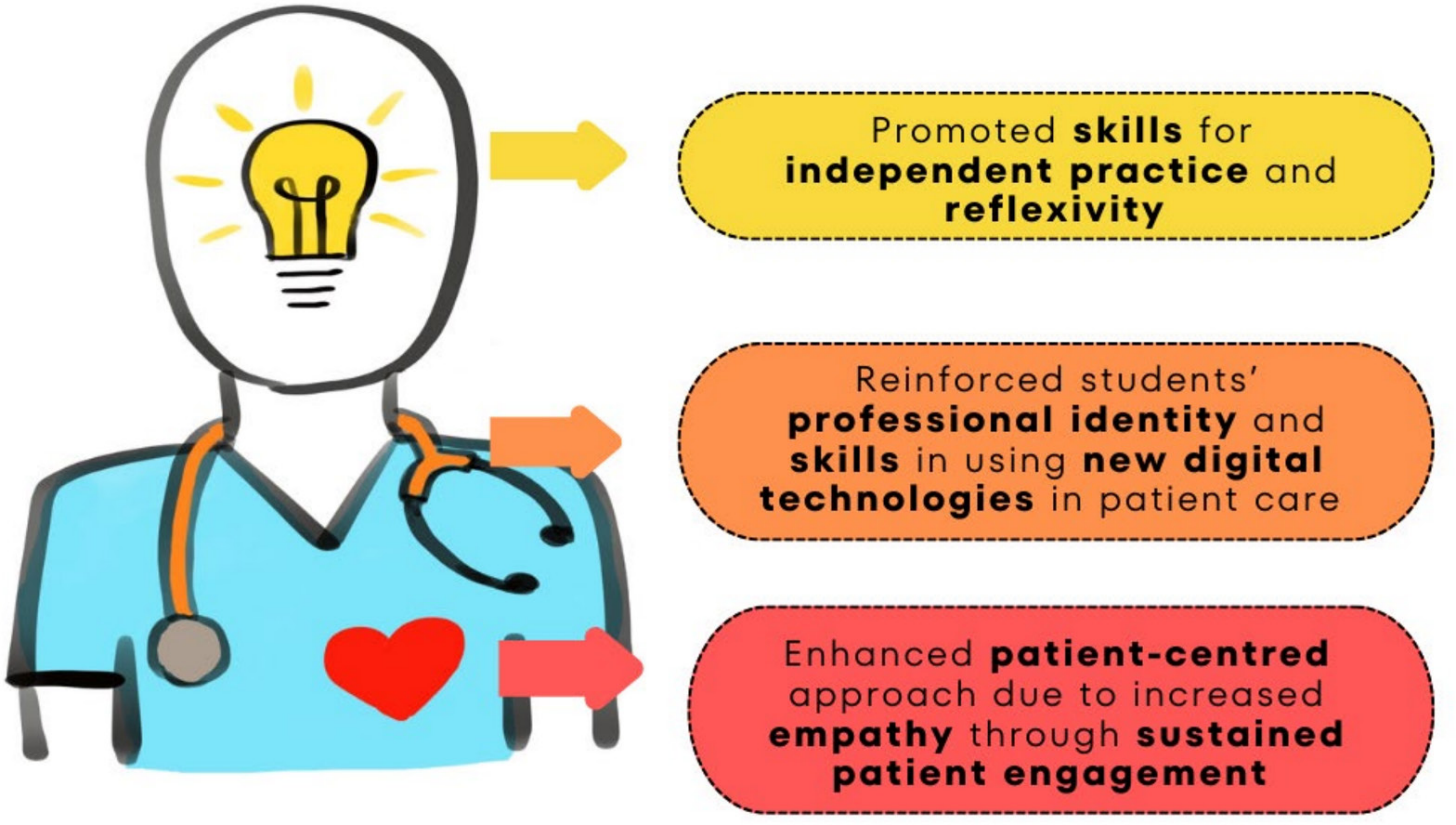
Common themes determined from student and tutor focus group data.

We determined three key themes following the data analysis of both focus groups. These are described in Figure 5.

#### 3.3.1 Increased Empathy Through Sustained Patient Engagement Enhances Patient‐Centred Approach

The greatest opportunity of the placement was that students felt that they viewed a patient as a person, allowing them to focus on building a personal connection rather than solely focusing on clinical aspects. The tutors also welcomed this aspect.

If you're forming rapport with them, then that means you're also creating, like, this sort of bond where, like, you're trying to understand your patient on a more human level and not just, like, on a doctor to patient level. (Student 3)

It was nice to see that the reasons why they [students] remembered them wasn't to do with any of the conditions that they had … recognising them as a person and not just a patient with a list of conditions or certain medications or certain surgeries. (Tutor 1)

The student participants appreciated the continuity of follow‐ups for a patient; the follow‐up session provided them with the opportunity to gain deeper insight into changes in their patient's health over time. Tutors recognised that students valued this element of the DCCP.

You learn more, like, from the patient because we rarely do, sort of like, learning sessions where we actually follow through on a patient. I think it's very interesting to see, like, you know … especially after, like, the few months gap on how they're doing now. And there was actually quite a lot. (Student 1)

Both students and tutors believed that students enhanced their empathy skills, allowing them to develop a deeper understanding of their patients.

I was kind of asking them about how they'd find it kind of connecting with the patient … one of the students actually said, ‘No … I feel like I've not got very much actually in common with this person, but I'd really valued that because now I understand them’. And I thought that was actually really very powerful, because that's actually what empathy is all about, is not it? And I thought that worked really well. (Tutor 2)

It's hard to go away from the session and not feel, to an extent, emotionally attached to your patient. (Student 3)

Students whose patient volunteers withdrew before the second session, and who were subsequently assigned new patients, quickly adapted to the challenge and reported gaining an understanding of both individuals. However, some learning opportunities may have been lost as a result.

#### 3.3.2 New Digital Technologies Reinforce Students' Professional Identity and Skills in Patient Care

Both student and tutor participants recognised that gaining experience with remote consulting platforms provided students with exposure to authentic and realistic clinical practice, despite encountering technical challenges.

[…] getting to speak to the patients through the Near Me platform was quite a good way to get into using that as well, because, obviously, we do use that a lot in actual practice. (Student 2)

I do think that it's valuable because it teaches some skills and communication like how to establish rapport with patients over digital media. (Tutor 2)

The students valued practising digital documentation as an authentic experience that reinforced professional identity; however, tutors may not have fully recognised its significance.

I really like the computer where we get to, like, make the patient's notes because that is my first time, you know, doing something like that myself. I've always just seen, like, you know, more senior doctors do that. (Student 1)

I'm not sure some of them, kind of, really understood what the purpose was. Is that, oh, we're just learning to put something on TrakCare, or is it the importance of the documentation? (Tutor 1)

Practising documentation enabled students to develop an understanding of continuity of care, including best practices for maintaining it and the essential skills required to effectively support it.

It's very much like the doctor's note, which I've never done before myself. And it gives me insight. It's, like, oh, so I … actually have to look back at my note before and then go off on that. (Student 1)

They understood the importance of that more when it came to the follow‐up consultation because they were relying on what they wrote three months ago to remind them of, I suppose, the specifics that they wanted to pick up on. (Tutor 1)

#### 3.3.3 Promotion of Independent Practice and Reflexivity

Tutors with debriefing experience encouraged students to ask more challenging questions, thereby enhancing their skills for independent clinical questioning.

We also had a conversation about avoidance and things that, [being students], they were avoiding asking or digging a little deeper because they didn't quite know what the response would be, or they didn't want to upset someone. (Tutor 1)

Tutors believed that having a second‐year student in the group facilitated the identification of previously unrecognised insights among the third‐year students, thereby enhancing their reflexivity.

Definitely notice the difference if you had a second year in there because they were really doing that kind of observation role … picking up on a lot of different things that the third years weren't necessarily … asking specific questions about. (Tutor 1)

Students believed that the placement could have had a greater impact if introduced in the earlier years of their training, given that engaging with authentic practice at an earlier stage may have encouraged independent practice sooner.

I would really benefit from having this sort of session in my second year or even, like, first year and having early exposure to how patient notes are being taken – how patient history is being taken. (Student 3)

## 4 Discussion

This study aimed to assess the acceptability of the DCCP and identify the challenges and opportunities associated with this alternative placement. A key finding of this study was the broad acceptability of the DCCP across stakeholders. Qualitative data further suggested that authentic engagement with digital healthcare technologies facilitated professional identity formation, while also revealing the unique benefits and challenges of remote consultation as a clinical learning environment.

A key finding of this study was the broad acceptability of the DCCP across stakeholders.

### 4.1 Acceptability Across Stakeholders

The DCCP was well received, as evidenced by high levels of perceived effectiveness and intervention coherence across all stakeholder groups [12]. There was a shared view that the DCCP successfully achieved its intended purpose but with challenges and perceived opportunities for stakeholders, which should be considered when delivering such learning sessions in the future. Overall, tutors showed the highest agreement across categories, while second‐year students demonstrated greater agreement than third‐year students. This finding is also reiterated in the focus groups, suggesting that this type of placement may be more suitable for early‐year medical students. Patient volunteers showed a higher percentage of *agree*/*strongly agree* responses for affective attitude, likely because this cohort comprised individuals who had chosen to volunteer in the project.

The DCCP successfully achieved its intended purpose but with challenges and perceived opportunities for stakeholders.

### 4.2 Exposure to Authentic Digital Healthcare Technology Can Affect Students' Professional Identity

Very few studies examine the acceptance of health technology systems, such as EHR, among early‐year medical students [23]. Combining longitudinal patient interactions, remote consultation and digital documentation using healthcare technologies created an authentic learning experience that reinforced students' independent practice and professional identity development. This occurred despite technical challenges, which inadvertently added to authenticity. This full‐cycle engagement, from remote interaction to digital documentation, provided students with a sense of real‐world responsibility and professional integration—which was valued by students within the DCCP.

This full‐cycle engagement, from remote interaction to digital documentation, provided students with a sense of real‐world responsibility and professional integration.

Previous studies [24] emphasise that students develop a stronger sense of professional identity when given responsibility and opportunities to manage entire clinical processes independently. Third‐year student participants, although expressing preference for such placements earlier in their training, demonstrated learning gains, particularly in digital documentation practices. This suggests that students assign significant value to hands‐on experience with authentic digital platforms, which aligns with previous studies confirming their preference for opportunities to engage with authentic digital platforms [25].

Across all stakeholder groups, neutral responses in the ethicality category may reflect the complexity of granting students access to patient medical records for authentic practical experience. Given the legal and ethical complexities associated with documentation of real patient medical records [26] for early‐year medical students, stakeholders may have preferred the training version of the digital documentation platform and still valued its authenticity, leading to neutral responses in this ethicality category.

### 4.3 Benefits and Challenges of Longitudinal Patient Contact via Remote Consultation

A longitudinal approach, in which students engage with a patient over time, enhances patient‐centredness among medical students [7]. The DCCP supported this enhancement despite the consultations being conducted remotely, likely owing to the sustained patient–student relationship established during the placement. Challenges remained: Consultations were perceived as too lengthy, some students had follow‐up consultations with new patient volunteers because of dropout and limited clinical change in some patient volunteers caused confusion about the follow‐up's purpose. Because comparative data with face‐to‐face placements were unavailable, whether these issues occurred less frequently in face‐to‐face placements remains unclear.

The DCCP supported this enhancement despite the consultations being conducted remotely, likely owing to the sustained patient–student relationship established during the placement.

Remote consultations are easier once a relationship has been established [27, 28]. However, the skills required for remote consultations differ from those for face‐to‐face interactions, with greater effort needed to establish rapport and trust in the former case [29], which necessitates targeted training of health professional students. The DCCP offers structured opportunities to develop these skills, which are essential for patient‐centred communication in digital healthcare.

### 4.4 Strengths and Limitations

#### 4.4.1 Strengths

We incorporated perspectives from all three stakeholder groups and utilised an established acceptability framework [12], supporting a validated, structured and systematic evaluation of participants' perceptions and experiences. This enhances the study's transferability to future studies and educational programme development. Three independent researchers with varied experience contributed to data collection and analysis process, iteratively reviewing and debriefing common themes, which supported the trustworthiness of the findings [30].

#### 4.4.2 Limitations and Recommendations

This study was limited by its small sample size, particularly in the qualitative component, which may restrict the depth and trustworthiness of the findings despite the satisfactory response rates. The novelty of the placement meant that no comparison group was available, requiring the use of an acceptability framework instead of comparative analysis. As a pilot study conducted with limited resources, the findings should be considered preliminary and are intended to inform future research. While assessing acceptability is a critical first step in intervention development [31], future research should also examine scalability, sustainability and comparability with existing medical education placements.

Additionally, second‐year students were not represented in the focus groups; however, their perspectives were partially captured through questionnaire data, which may not fully reflect the breadth of their experiences. Participation bias is also possible, as individuals with particularly positive or negative experiences may have been more likely to participate.

## 5 Conclusion

Overall, this study demonstrates that the DCCP—a novel early‐year, longitudinal digital placement integrating remote consultations and electronic documentation—was acceptable across stakeholder groups despite implementation challenges. These preliminary findings suggest that early exposure to this placement may support the development of patient‐centred care skills and better prepare students for technology‐enabled healthcare practice. Future research should use larger, purposively sampled cohorts and comparative designs to evaluate the scalability and sustainability of such placements within medical curricula.

## Author Contributions

**Tina Huang:** conceptualization, investigation, methodology, funding acquisition, writing – original draft, writing – review and editing, data curation, project administration, formal analysis. **Anita Laidlaw:** conceptualization, writing – review and editing, supervision, methodology, formal analysis. **Rachel Falconer:** investigation, writing – review and editing, formal analysis. **Rona Patey:** conceptualization, supervision.

## Funding

This project was awarded the ASME GMC Medical Education Excellence Award 2024. The funder had no role in study design, data collection, analysis, interpretation or manuscript preparation. Dr Tina Huang's post, as the Digital Consultation Project Lead, was funded through the Medical Additional Costs of Teaching (ACT) scheme, provided by the Scottish Government, which supports undergraduate medical education activities within NHS Scotland.

## Ethics Statement

This study was approved by the School of Medicine, Medical Sciences and Nutrition Ethics Review Board at the University of Aberdeen (ref ID 4574365).

## Conflicts of Interest

The authors declare no conflicts of interest.

## Supporting information


**Appendix S1:** Replication framework for the DCCP.
**Appendix S2:** Tutor focus group questions.
**Table S1:** Student questionnaire responses.
**Table S2:** Tutor questionnaire responses.
**Table S3:** Patient volunteer questionnaire responses.

## Data Availability

The data that support the findings of this study are available from the corresponding author upon reasonable request.
