# Supplementary material for: Acceptability of a Pilot Digital Longitudinal Consultation Clinical Placement for Early‐Year Medical Students, Tutors and Patients
Source: Clin Teach. 2026 Sep 15;23(5):e70526. doi: 10.1111/tct.70526 (PMC13577801; doi:10.1111/tct.70526)
Supplement: Supplementary file 1 [file TCT-23-e70526-s001.docx]

**Appendix 1**

Replication framework for the DCCP:

| **Component** | **Description** |
| --- | --- |
| Group composition | Two third-year medical students and one second-year medical student per group |
| Patient involvement | One patient volunteer with a long-term condition per group |
| Consultation format | Single 30-minute consultation conducted remotely |
| Consultation focus | Exploration of the impact of illness or impairment on the patient’s and family’s daily life (e.g. work, social activities, routines) |
| Student roles | Third-year students lead the consultation; second-year student observes |
| Documentation | Consultation summary completed post-consultation using Training TrakCare or written format |
| Data environment | Training TrakCare environment; no real patient data accessed |
| Patient confidentiality | No patient-identifiable information recorded |
| Faculty involvement | One clinical tutor facilitates a one-hour debrief with two groups |
| Feedback mechanism | Patient volunteer feedback collected to support student learning |

**Appendix 2**

**Tutor Focus Group Questions**

| Questions (Direct from transcription) | Acceptability Framework Domain(s) |
| --- | --- |
| - Did you get any impression of the impact this project had on patient volunteers? | Affective attitude  Ethicality |
| - Was there anything unexpected that came up in the debriefs? - How did you find managing the logistics of this type of remote consultation? - Do you think DCCP sessions could run without a project lead [in addition to tutors]? | Burden  Opportunity costs |
| - Do you think teaching students skills for remote patient consultation is important for their future careers? | Ethicality |
| - What were the main themes that were coming out during the debriefs? - Do you think students understood what we were trying to get them to document? | Intervention coherence |
| - How well do you think the DCCP achieved the intended aims of the project? - Did you feel there was enough time allocated for each session? - I understand there were some student absences, do you think that had an impact on how the sessions ran on the day? - When the same patient was not available for both sessions, did you feel that had an impact on the intended learning and if so, how? | Perceived effectiveness |
| - Did you feel confident and able to troubleshoot challenges which came up? - How did you perceive the dynamic of having students from different year groups working together? | Self-efficacy |

**Student Focus Group**

| Questions (Direct from transcription) | Acceptability Framework Domain(s) |
| --- | --- |
| - How did you find taking part in the DCCP? - What were your thoughts about the debrief? | Affective attitude |
| - Did you feel there was enough time allocated to the various tasks? - Did you experience any technical difficulties with the electronic platform? - Were there any particular challenges or difficulties that came up during either of the sessions? - If you experience any technical difficulties during the sessions, did you feel the tutors were able to troubleshoot and address these? | Burden |
| - Is there anything specific you took away from this experience that you think will have an impact on how you view or interact with patients in future? - Did you feel any emotional impact from speaking with patients and if so, how was this handled within the debrief? - What are your reflections now, compared to immediately after the session? Have these changed? | Ethicality |
| - What did you understand to be the main aims of the project? - How did you find the experience of taking charge of a consultation without a tutor directly supervising you in the room? - Do you think it was particularly helpful to practice electronic documentation on TrakCare? - Were there topics which came up during the patient interviews which you felt you did not know how to approach with the patient? - Did you feel as a group you were able to navigate through these? - Do you think learning skills to manage a remote consultation provided additional challenge? - Do you think the debrief was helpful? - Was it useful to hear other groups experiences? - How did you feel being in a group with different year groups? | Intervention coherence |
| - How did you find coming to a University building to undertake a remote consultant rather than doing this from home? - Are you aware of any challenges or barriers to students taking part in these sessions? - There were quite a number of student absences - do you have any suggestions for ways to improve student engagement? | Opportunity costs |
| - Do you feel the opportunity to follow the same patient over a period of time was useful? - Did this feel like a different type of patient encounter to those that you have had in the curriculum to date? - Do you think this type of session should be delivered to more students? | Perceived effectiveness |
| - Do you think this placement has impacted how you view the skills needed for remote consultations? - Did you find it beneficial to practice documenting a consultation? | Self-efficacy |

**Appendix 3**

Table 1. Student Questionnaire Responses

| **Component** | **Student Questionnaire Responses** | | |
| --- | --- | --- | --- |
|  | | **Percentage of Agree/ Strongly Agree responses** | |
|  | **Likert Scale Questions** | **Y3 (n=9)** | **Y2 (n= 6)** |
| Affective attitude | I enjoyed taking part in the Digital Consultation Clinical Placement. | 56% | 83% |
| Burden | It was easy to take part in the consultations using the Near Me platform. | 100% | 67% |
| Burden | I would have liked further support during the consultation with the patient.* | 89% | 50% |
| Ethicality | I was comfortable discussing the patients’ medical and social history in video call format. | 100% | 100% |
| Ethicality | I would have preferred if my group’s clinical summary note had been reviewed by a clinician familiar with my case.* | 33% | 50% |
| Intervention coherence | I understood the purpose of the Digital Consultation Placement. | 63% | 100% |
| Opportunity costs | The time commitment required was reasonable, given my other academic responsibilities. | 50% | 67% |
| Perceived effectiveness | This placement did NOT provide me with a deeper insight into patient experiences.* | 67% | 83% |
| Perceived effectiveness | This placement improved my understanding of how non-medical factors can affect one’s health. | 78% | 100% |
| Perceived effectiveness | I found it beneficial to write a clinical summary using TrakCare. | 89% | 50% |
| Self-efficacy | I did NOT feel confident in my ability to consult with the patient.* | 89% | 100% |
|  | **Questions written in negative: Disagree was the desired response; therefore, these were the percentage of disagree and strongly disagree responses* | | |
| Overview | | | |
|  | This placement prepared me for future clinical placements. | 67% | 67% |
|  | | **Percentage of Yes responses** | |
|  | **Yes/No Questions** | **Y3** | **Y2** |
| Burden | Did you experience any challenges in using the technology?* | 56% | 67% |
| Perceived effectiveness | Did you find having the second consultation with the patients to be useful? | 55% | 83% |
|  | **Questions written in negative: No was the desired response; therefore, these were the percentage of No responses* | | |
| Overview | | | |
|  | This type of clinical placement would be beneficial for all 2nd- and 3rd-year medical students. | 63% | 83% |

Table 2. Tutor Questionnaire Responses

| **Component** | **Tutor Questionnaire Responses** | |
| --- | --- | --- |
|  | **Likert Scale Questions** | **Percentage of Agree/ Strongly Agree responses (n=4)** |
| Affective attitude | I enjoyed being a tutor in the Digital Consultation Clinical Placement. | 75% |
| Burden | The workload was manageable in terms of supporting and coordinating the students. | 100% |
| Ethicality | I felt the patients’ privacy and confidentiality were adequately protected during the consultations. | 100% |
| Ethicality | I would have preferred it if the students had consulted with patients whose medical history I am familiar with.* | 25% |
| Intervention coherence | I understood my role in supporting the students during the Placement | 100% |
| Opportunity costs | The time commitment required was reasonable, given my other professional responsibilities. | 100% |
| Perceived effectiveness | This placement did NOT provide the medical students with a deeper insight into patient experiences.* | 75% |
| Perceived effectiveness | This placement improved medical students’ understanding of how non-medical factors can affect one’s health. | 100% |
| Self-efficacy | I felt confident in my ability to debrief the students and facilitate their learning during the Placement. | 100% |
|  | **Questions written in negative: Disagree was the desired response; therefore, these were the percentage of disagree and strongly disagree responses* | |
|  | **Yes/No Questions** | **Percentage of Yes responses** |
| Burden | Did you encounter any challenges with the logistics or technology during the sessions?* | 50% |
| Perceived effectiveness | Did you find it beneficial for the students to have a second consultation with the patient? | 50% |
| Perceived effectiveness | Did your debriefing sessions contribute to the students’ learning and help them reflect on their consultation experiences? | 100% |
|  | **Questions written in negative: No is the desired response; therefore, these were the percentage of No responses* | |
| Overview | | |
|  | This type of clinical placement would be beneficial for all 2nd- and 3rd-year medical students. | 75% |
|  | Would you be willing to participate as a regular tutor for the Digital Consultation Placement? | 100% |

Table 3. Patient Volunteer Questionnaire Responses

| **Component** | **Patient Volunteer Questionnaire Responses** | |
| --- | --- | --- |
|  | **Likert Scale Questions** | **Percentage of Agree/ Strongly Agree responses (n=14)** |
| Affective attitude | I enjoyed speaking with the medical students during the consultations. | 93% |
| Affective attitude | I was NOT comfortable sharing my experiences and story with medical students.* | 100% |
| Burden | It was easy to take part in the consultations using the Near Me platform. | 76% |
| Ethicality | My personal information and privacy were respected throughout the Digital Learning Experience. | 93% |
| Ethicality | I would have preferred it if the medical students had access to my medical records during the Digital Learning Experience.* | 21% |
| Intervention coherence | I understood the purpose of the Digital Learning Experience. | 93% |
| Intervention coherence | I would have liked to receive more information about the Digital Learning Experience beforehand.* | 64% |
| Opportunity costs | The time commitment required was reasonable. | 93% |
| Opportunity costs | I felt confident in my ability to share my experiences with the medical students. | 93% |
| Perceived effectiveness | I believe that the medical students benefited from interacting with me during the consultations. | 86% |
| Perceived effectiveness | My contribution did NOT aid medical students’ understanding of patient experiences.* | 93% |
| Perceived effectiveness | My contribution helped improve their understanding of how non-medical factors can affect one’s health. | 79% |
| Self-efficacy | I was able to share my experiences in the same way I would during a face-to-face consultation. | 86% |
|  | **Questions written in negative: Disagree was the desired response; therefore, these were the percentage of disagree and strongly disagree responses* | |
|  | **Yes/No Questions** | **Percentage of Yes responses** |
| Burden | Did you experience any challenges in using the technology?* | 85% |
| Opportunity costs | Did participating in the Digital Learning Experience prevent you from doing other activities that were important to you?* | 100% |
| Perceived effectiveness | Did you learn anything about your own health during the experience? | 21% |
| Perceived effectiveness | Did you find having the second consultation with the medical students to be useful? | 100% |
|  | **Questions written in negative: No was the desired response; therefore, these were the percentage of No responses* | |
| Overview | | |
|  | I would be willing to participate in the Digital Learning Experience in the future. | 100% |
|  | Have you previously participated in the University of Aberdeen’s patient partner program? | 71% |
